# Placental Dysfunction in Assisted Reproductive Pregnancies: Perinatal, Neonatal and Adult Life Outcomes

**DOI:** 10.3390/ijms23020659

**Published:** 2022-01-08

**Authors:** Claudio Manna, Valentina Lacconi, Giuseppe Rizzo, Antonino De Lorenzo, Micol Massimiani

**Affiliations:** 1 Department of Biomedicine and Prevention, University of Rome Tor Vergata, Via Montpellier 1, 00133 Rome, Italy; claudiomanna55@gmail.com (C.M.); valelcc@gmail.com (V.L.); giuseppe.rizzo@uniroma2.it (G.R.); delorenzo@uniroma2.it (A.D.L.); 2 Biofertility Center of Reproductive Medicine, Viale degli Eroi di Rodi 214, 00128 Rome, Italy; 3 Saint Camillus International University of Health Sciences, Via di Sant’Alessandro 8, 00131 Rome, Italy; 4 Department of Obstetrics & Gynecology Moscow Russian Federation, The First I.M. Sechenov Moscow State Medical University, Trubetskaya Street, House 8, Building 2, 119991 Moscow, Russia

**Keywords:** placenta, placental dysfunction, assisted reproduction techniques, infertility, preeclampsia, intrauterine growth restriction, trophoblast invasion, PlGF, sFLT-1, EGFL7

## Abstract

Obstetric and newborn outcomes of assisted reproductive technology (ART) pregnancies are associated with significative prevalence of maternal and neonatal adverse health conditions, such as cardiovascular and metabolic diseases. These data are interpreted as anomalies in placentation involving a dysregulation of several molecular factors and pathways. It is not clear which extent of the observed placental alterations are the result of ART and which originate from infertility itself. These two aspects probably act synergically for the final obstetric risk. Data show that mechanisms of inappropriate trophoblast invasion and consequent altered vascular remodeling sustain several clinical conditions, leading to obstetric and perinatal risks often found in ART pregnancies, such as preeclampsia, fetal growth restriction and placenta previa or accreta. The roles of factors such as VEGF, GATA3, PIGF, sFLT-1, sEndoglin, EGFL7, melatonin and of ART conditions, such as short or long embryo cultures, trophectoderm biopsy, embryo cryopreservation, and supraphysiologic endometrium preparation, are discussed. Inflammatory local conditions and epigenetic influence on embryos of ART procedures are important research topics since they may have important consequences on obstetric risk. Prevention and treatment of these conditions represent new frontiers for clinicians and biologists involved in ART, and synergic actions with researchers at molecular levels are advocated.

## 1. Introduction

There is a rapidly increasing interest in placentation in obstetric adverse outcome, neonatal and adult life health after infertility, and its related therapies. Abnormal placentation is a common finding in the infertile population, even among those couples conceiving spontaneously after a period of infertility. A higher risk of preterm birth (PTB) and low birth weight (LBW) has been found in these pregnancies [1]. Moreover, abnormal placentation and obstetric complications such as PTB, preeclampsia (PE), and fetal growth restriction (FGR) have been associated with endometriosis, a common factor of infertility [2]. Another maternal condition at risk for the development of placental anomalies, commonly associated with infertility, is polycystic ovary syndrome (PCOS), in which the prevalence of gestational diabetes mellitus (GDM) is significantly increased [3]. Of note, GDM is an independent risk factor for the onset of placental disorders with altered structure, function and hypertrophic growth of the organ [4].

An important actual focus in reproductive medicine, neonatology and population health is that up to 6% (range between 0.2% and 6.4%) of European births is conceived by assisted reproductive technology (ART) [5] and concerns existing health conditions of individuals born following ART. ART is a group of in vitro techniques used to treat moderate and severe infertility, including in vitro fertilization (IVF), intracytoplasmic sperm injection (ICSI), frozen embryo transfer (FET), oocyte donation (OD), blastocyst culture, intrauterine insemination, and preimplantation genetic testing for aneuploidy (PGT-A). Unfortunately, each of these techniques may represent a possible confounding factor to the identification of a precise relationship between ART procedures and obstetric or neonatal outcomes, including children and their adulthood health. Many studies report an evident increase of obstetrical risk and perinatal complications with ART, especially for hypertensive disorders of pregnancy (HDP), from gestational hypertension to eclampsia [6,7,8,9,10,11,12,13]. A recent meta-analysis of 50 cohort studies including 161,370 ART and 2,280,241 spontaneously conceived singleton pregnancies found increased risks for several obstetric complications. Significant worst outcomes regarded pregnancy-induced hypertension, placenta previa, abruption, antepartum hemorrhage, oligohydramnios, cesarean delivery, PTB, very low birth weight (VLBW), LBW and perinatal mortality and morbidity [14].

The role of the placenta in these conditions has been demonstrated in several studies. It is well documented that ART can be associated with changes in placental morphology and structure, growth dynamics, imprinted and non-imprinted genes, and other aspects regulating placentation [15]. Several studies demonstrate that incidence of placenta previa was significantly higher in ART than in spontaneous conception (OR 3.14, 95% CI [8]; RR 3.71, 95% CI [16]). Moreover, the placentas from ART pregnancies presented a significantly greater weight and higher placental weight-to-birth weight ratio [17,18]. Some observations indicate that placentas of pregnancies obtained by ART have an increased placental thickness and higher incidence of hematomas [19]. It has been also demonstrated that in children conceived by ART the frequency of imprinting disorders is higher than expected in the general population. ART is associated with increased risk of epigenetic alterations influencing gene expression and DNA methylation in early development and in the placenta, eventually triggering diseases and affecting long-term health [20,21,22]. These findings support the hypothesis of a primary ART responsibility for adverse perinatal outcomes with evident suspicions for anomalies of placentation, although fertility treatments themselves are often associated with impaired placentation and related adverse pregnancy outcomes [1]. Infertile couples who did not undergo ART showed an increased risk of adverse obstetric outcomes, such as placental abruption, fetal loss and GDM [23].

Based on these observations, the main question to address is whether placental alterations (from macroscopic to molecular level) observed in treated infertile patients are the result of ART or if they originate from infertility itself. A deeper investigation of the underlying molecular basis of reduced placental functions in infertility condition and especially after ART treatment is then needed.

In this review, we provide a summary of the most recent literature on placental dysfunction associated with obstetric, perinatal outcomes in singletons born after NON-ART and ART treatments. ART and infertility may be the cause of common dysregulated pathways, including changes in trophoblast invasion, environmental conditions, vascular defects, chronic inflammation, and oxidative stress. Each of them can be prevalent or coexist with others with different and common molecular pathways in a grading and timing that can configurate many clinical features, leading often to neonatal–adult and maternal consequences. This review is based on systematic reviews (SRs), large cohort studies, meta-analyses, and genetic, epigenetic and molecular studies.

## 2. Abnormal Placentation and ART: Molecular Factors and Involved Signaling

Abnormal placentation may present in a variety of phenotypes, severity, clinical conditions and consequences as the result of several types of infertility treatments and techniques used in ART. For these reasons, it becomes difficult to linearly relate placental influence to obstetrical and perinatal (or neonatal) outcomes after ART.

**Figure 1:**
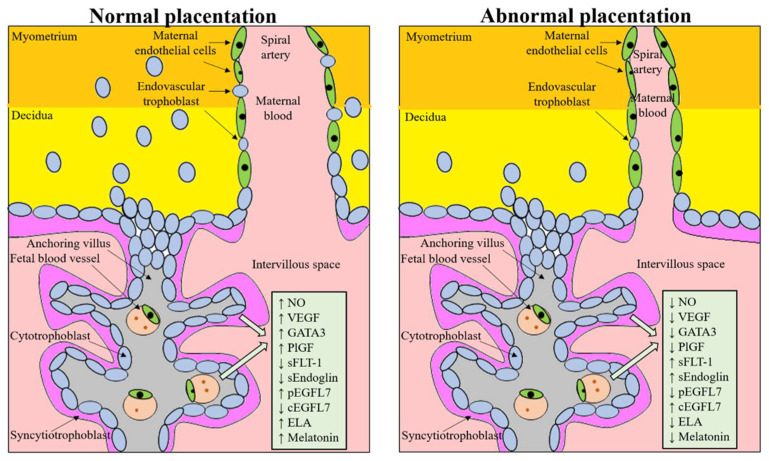
Molecules regulating proper placental development, whose dysregulation is involved in an abnormal placental development. NO, nitric oxide; VEGF, vascular endothelial growth factor; GATA3, GATA binding protein 3; PlGF, placental growth factor; sFLT-1, soluble fms-like tyrosine kinase 1; sEndoglin, soluble endoglin; pEGFL7, placental epidermal growth factor-like domain 7; cEGFL7, circulating epidermal growth factor-like domain 7; ELA, elabela.

An altered expression of factors and molecules involved in proper placental development, leading to impaired trophoblast invasion and subsequent reduced vascular remodeling and placenta hypoperfusion, sustain several clinical conditions leading to obstetric and perinatal risks often found in ART pregnancies, such as PE, FGR and placenta previa or accrete (Figure 1) [24,25,26,27,28]. Syncytiotrophoblast stress has been associated with a dysregulated expression of placental growth factor (PlGF) and soluble fms-like tyrosine kinase 1 (sFLT-1) [29,30]. Circulating levels of the anti-angiogenic factor sFLT-1 are increased, and those of PlGF are decreased even before the onset of the clinical symptoms of PE and FGR [31,32,33,34,35]. The increased ratio sFLT-1:PlGF is thought to contribute to the systemic endothelial response and correlate with the severity of FGR and PE. A recent study indicates that the release of sFLT-1 from the placenta is regulated by the epidermal growth factor receptor (EGFR) pathway and the mitochondrial electron transport chain and its downstream pathways, both significantly increased in preeclamptic placentas [36]. The inhibition of these signaling pathways significantly reduces sFLT-1 release from primary cytotrophoblast cells [36]. Vrooman demonstrated in the rat model that an embryo culture from the 1-cell to blastocyst stage increased levels of sFLT-1 together with placental overgrowth, reduced fetal weight, and lower placental DNA methylation [37].

Over the last decades, several other factors have been demonstrated to be altered in pregnancy disorders associated with abnormal placentation (e.g., soluble endoglin (sEndoglin), PlGF and epidermal growth factor-like domain 7 (EGFL7)), with the aim to create a panel of markers to allow an earlier and more precise diagnosis of PE. sEndoglin has been shown to be upregulated in abnormal placenta, typical of FGR and PE, and released into the maternal blood, where it acts as antiangiogenic factor by inhibiting transforming growth factor-beta (TGF-β) signaling in the vasculature. sEndoglin markedly increased, beginning 2 to 3 months before clinical manifestations of PE [38,39]. EGFL7, originally discovered as a largely endothelial restricted gene, has been recently demonstrated to be expressed in the placenta and involved in placental angiogenesis and trophoblast migration [40,41,42]. Altered EGFL7 expression is associated with abnormal placentation and systemic maternal endothelial dysfunction, observed in PE [40,43,44]. Maternal treatment with nitric oxide (NO) donors increases placental EGFL7 levels and improves maternal hemodynamic state and perinatal outcome [45,46]. In the maternal circulation, endothelial dysfunction and abnormal hemodynamic state are associated with increased levels of EGFL7, which return to control levels after NO treatment [44]. Moreover, EGFL7 dosage in maternal circulation allows to discriminate between PE and FGR [43]. Although there are no data correlating EGFL7 with ART, dosage of its circulating levels could help identify ART-associated abnormal placentation.

In FGR and placental insufficiency, the levels of the hormone melatonin are significantly reduced, and this decrease is correlated with proinflammatory activities of tumor necrosis factor alpha (TNF-α), interleukine-1beta (IL-1β), and IL-6 [47]. Melatonin is an antioxidant factor and an anti-inflammatory agent [48,49]. Several studies support the production of this hormone in the ovary as a whole [50,51], the granulosa cells, including those making up the cumulus oophorus [52,53], and the oocyte [54]. In women undergoing ART, melatonin significantly increased the implantation rate [55]; a similar result was obtained in women affected by PCOS undergoing intrauterine insemination [56]. Melatonin crosses the cell membrane, thus interacting with intracellular molecules via different signaling pathways and displaying scavenger functions [57]. Melatonin upregulates the primary implantation receptors, ErbB1 and ErbB4, and significantly reduces intracellular ROS in mouse blastocysts (increasing the embryo total antioxidant capacity) and promotes mitosis of the inner cell mass and trophectoderm cells [58]. Recently, it has been demonstrated that the supplementation of culture medium with melatonin (10^−9^ mol/L) improves the growth of mouse parthenogenetic embryo potentially by promoting cell cycle progression [59]. Despite all these functions, melatonin is not present in culture media used in ART. We could speculate that the addition of melatonin to the ART media may be beneficial.

## 3. Pathophysiology of the Placenta in Pregnancy Complications and ART Pregnancies

Altered hormonal milieu, epigenetic changes, immune activity dysregulation, dysmetabolism and inflammation are all conditions attributable to ART and associated with an abnormal placentation. This section provides a theoretical basis on placental changes caused by ART that are most strongly associated with an increased risk of fetal, neonatal and long-term diseases.

### 3.1. Altered Hormonal Milieu Effect on Placental Development

The interpretation of the effects of ARTs on pregnancy and the possible associated pathologies is complicated by the supra-physiological hormonal levels in the recipient, as a consequence of the ovarian stimulation treatment [37]. Bourgain and Devroey in 2003 suggested that increased hormone blood levels might alter the timing of endometrial receptivity, with possible suboptimal embryo implantation and development [60]. It was also suspected, by studies in animal models, that high estrogen levels exert a detrimental effect on spiral artery remodeling by the trophoblast [61]. Moreover, it has been observed that, in FET procedures, levels of progesterone higher than 32.5 ng/mL on the day of embryo transfer, were associated with no pregnancy [62]. This cut off level seems improbable in ovarian controlled stimulation conditions of fresh IVF cycles, where many corpora lutei are functioning and progesterone level, together with estradiol, are much higher. Conversely, the lack of hormones might also affect placental development by creating conditions leading to increased risks for HDP, as it has been suggested for FET following hormonal endometrial preparation [63,64]. Using these protocols, ovulation does not occur, and the corpus luteum, which secretes important protective vasoactive substances, is not formed [9,10,65]. Among these factors, relaxin is a potent vasodilator promoting vascular compliance [66] and facilitating the adaptation of the maternal cardiovascular system to pregnancy [67]. Moreover, in programmed FET cycles, the use of estradiol and progesterone has been recently associated with obstetric adverse outcomes, i.e., PTB, LBW, and PE, which are small for gestational age (SGA) and large for gestational age (LGA) when compared to natural FET cycle [9]. Thyroid hormone (TH) has been also demonstrated as an important player in reproduction; both hypothyroidism and hyperthyroidism have been associated with subfertility, recurrent miscarriages, and adverse pregnancy outcomes [68,69]. It has been recently demonstrated that TH regulates protease expression and activation of Notch signaling in implantation and embryo development [70]. Moreover, levothyroxine (LT4) treatment has a positive effect on conception capacity and reduces miscarriages when administered to euthyroid women with autoimmune thyroid disease affected by recurrent miscarriages [71].

Several common infertility conditions, such as endometriosis and PCOS, often treated with ART, are characterized by a hormonal dysregulation that affects proper placentation. In endometriosis, a defective deep placentation may derive from functional abnormalities of the eutopic endometrium, as well as an imbalance in endocrine and inflammatory markers [72]. PTB, placenta previa, placental abruption, gestational hypertension, PE, LBW, SGA, cesarean delivery, postpartum hemorrhage, and stillbirth have been significantly associated with endometriosis in a systematic review and meta-analyses, including in 39 studies [73]. PCOS is another condition leading to infertility, in which the defective trophoblast invasion and placentation may be caused by mother’s hyperandrogenism [74,75]. Testosterone can act directly on trophoblast invasion, with modifications of placenta morphology and function [76,77]. PCOS is a chronic low-grade inflammation associated with metabolic dysfunction that is enhanced in pregnancy by the induction of an endothelial dysfunction [78]. This condition might in turn prevent normal remodeling of spiral vessels and the physiologic decrease of uterine artery impedance, thus reducing the depth of endovascular trophoblast invasion. As a consequence of these patterns, in patients with PCOS the placental weight, thickness, density and volume was found significantly reduced [76]. In women with PCOS undergoing IVF, the risk of adverse pregnancy outcomes was found to be significantly elevated. In a recent meta-analysis including 29 observational studies, Sha et al. (2019) described an increased risk for GDM, pregnancy-induced hypertension (PIH), LGA, miscarriage and PTB in women with PCOS undergoing ART [79].

### 3.2. Epigenetic Changes after ART Techniques Are Associated with Altered Gene Expression in the Placenta and Congenital Imprinting Disorders

Epigenetic regulatory mechanisms can originate from several sources: direct DNA methylation, non-coding RNA, imprinting, and post-translational modification of histone proteins and chromatin remodeling [80]. According to some clinical studies, it was speculated that prolonged exposure to extracorporeal environment might predispose human embryos to disorders of genomic imprinting and to epigenetic modification [81]. Each stage of embryo development, from fertilization to blastocyst formation, also in humans, can undergo to epigenetic changes and consequent differential gene expression in the placenta [82,83]. In the pre-implantation period, the embryonic epigenome is entirely reprogrammed by specific methylations [84], leading to an increase in the risk of gene expression alterations under specific circumstances. For example, there is evidence that aberrant fetal DNA methylation might cause abnormal development of the placenta and recurrent spontaneous abortion [85].

**Figure 2:**
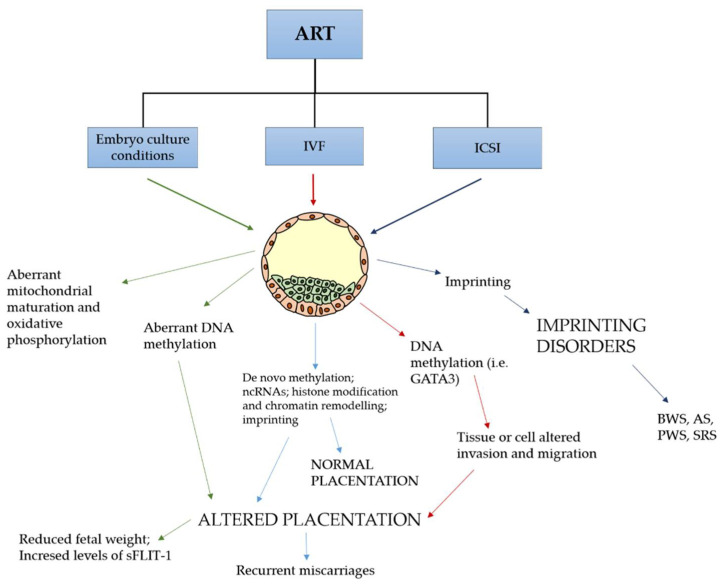
ART techniques affecting epigenetics in the pre-implantation embryo. Epigenetic dysregulation is involved in an abnormal placentation and genetic imprinting disorders. sFLT-1 denotes soluble fms-like tyrosine kinase 1, GATA3 GATA binding protein 3, BWS Beckwith–Wiedemann syndrome, AS Angelman syndrome, PWS Prader–Willi syndrome, SRS Silver–Russell syndrome.

We must consider that ART procedures occur simultaneously to this extensive epigenetic reprogramming and we cannot rule out that the stress involved in ART might affect the establishment and/or maintenance of genomic imprinting (Figure 2). Actually, more than 100 imprinted genes have been found to be strictly involved in the growth and development of embryos [15]. In placentas from IVF pregnancies, a different methylation pattern was observed in comparison to non-IVF gestations and environmental conditions were considered responsible for these findings (Figure 2) [82,83]. IVF disturbs the DNA methylation of the imprinting control region of *H19/Igf2*, *Snrpn*, *Peg3*, and *Kcnq1ot1* genes, inducing morphological alterations in the placenta and an increased risk of adult metabolic syndrome [86]. Studies using animal models have demonstrated a dysregulation of several maternally and paternally expressed imprinted genes (e.g., *Kcnq1ot1* involved in placental growth, *Peg10* that is required for the differentiation of placental spongiotrophoblast and labyrinth, *Peg11/Rtl1* involved the development of placental labyrinth and nutrient passive transport, *Sfmbt2* playing a key role in the maintenance of trophoblast cell types) in the IVF group as compared to the control group [87,88,89]. Most of these genes play an important role in the proper placental morphology and function. Moreover, specific transcription factors, such as GATA binding protein 3 (GATA3), produced by trophectoderm at blastocyst stage, may be responsible for tissue or cell altered invasion and migration under the influence of the local environment [90].

Consequently, particular attention was paid to blastocyst stage transfer and its possible adverse effect on perinatal outcome. In this regard, there are data from animal studies demonstrating that in vitro fertilization and embryo culture can strongly impact the placental transcriptome [91,92]. In a multicenter double-blind randomized controlled trial, including 836 couples, a significative reduction of birthweight (158 g) and an increase of PTB (8.6% vs. 2.2%) was associated with one of the two culture media used for embryo culture [93]. Moreover, it has been demonstrated that in vitro culture conditions may obstacle mitochondrial maturation and oxidative phosphorylation, inducing cells to increase glycolysis with the aim of maintaining energetic homeostasis: this pathway is represented by the so-called “Warburg effect” [94]. This adaptation pathway may induce epigenetic changes with possible adverse consequences on fetal growth (Figure 2) [94]. There is evidence that the longer the in vitro embryo culture lasts (i.e., blastocyst transfer in comparison to cleavage stage), the more epigenetic changes that can occur [81,94], especially in the developing placenta, although this is not surprising. In this respect, we should consider that this organ develops from the outer trophoblastic layer and consequently is more sensible to epigenetic regulatory changes caused by environmental manipulation and influences during ART. Placental overgrowth was demonstrated by Vroman in the rat model, where embryo culture from 1-cell to blastocyst stage showed adverse outcomes. Reduced fetal weight and lower placental DNA methylation, together with the abovementioned increased levels of sFLT-1, were observed [37]. Additional evidence of aberrant DNA methylation following in vitro embryo culture has been obtained using the bovine model [95]. In this study, 2-, 8- and 16-cell stage embryos were flushed from the oviducts and allowed to further develop in vitro up to the blastocyst stage and their methylome investigated. It was clear that each step of the in vitro culture gave a contribution to epigenetic alterations and that the highest levels resulted around the time of early genomic activation of embryo. In this regard, a recent discovery demonstrated that human embryonic genomic activation initiates at one-cell stage [96].

However, it would be important to distinguish the potential contribution of epigenetic problems related to infertility from epigenetic alteration deriving from the culture conditions [97]. Besides the ART procedures themselves, differential methylation has also been identified in placentas of pregnancies conceived from couples with infertility utilizing different fertility treatments [82,98]. Differential DNA methylation in placentas from pregnancies conceived with different fertility treatments, even as early as the late first trimester, has been described [99].

Furthermore, ART can influence epigenetic regulation of placental formation and function by changing the embryonic environment, placental gene expression, and placental adaptive response to embryonic development [100]. In this respect, Zhang et al. [91] analyzed genes in placental samples obtained from the IVF and control groups, identifying seventeen upregulated and nine downregulated genes. Moreover, when compared to normal pregnancies, the transcriptome profile of first-trimester placental villi appeared significantly altered in IVF-ET pregnancies. A total of 3405 differentially regulated genes in the placenta following IVF (>2-fold change; *p* < 0.05) was identified by Zhao et al. [92], with 1910 upregulated and 1495 downregulated. The study revealed that these genes are involved in more than 50 biological processes and pathways: coagulation cascades, immune responses, transmembrane signaling, metabolism, cell cycle, stress control, invasion, and angiogenesis, all playing an important role in early pregnancy. From a clinical point of view, many studies have provided useful data on placental pathologies linked to the in vitro environment in which embryo develops. A Swedish population-based registry study from 2002 through 2013 showed an increased risk of placenta previa after blastocyst transfer compared to cleavage-stage transfer (OR 2.18; 95% CI 1.79–2.65) and spontaneous conception (OR 6.38; 95% CI 5.31–7.66) [101]. For FET, higher birthweight, and higher risk of LGA and VLGA were found in blastocyst vs. cleavage stage transfer [102,103,104]. Similar data were reported by Huang (2020), comparing results from day 3 vs. day 7 embryo in 4489 infertile women from 2006 to 2017 [105].

An example of the invasiveness of the in vitro reproductive machinery is the introduction in the clinical practice of the ICSI, which overcomes severe male infertility problems [106], injecting a single sperm into the cytoplasm of a mature oocyte. The mechanical introduction of one sperm escaping the natural control of the fertilization process by the oocyte has raised several criticisms. Reasonable concerns were expressed considering that animal studies on the safety of this practice were limited in comparison with those performed by Edwards before Louise Brown was born in 1978. The revision of some large experience and reviews did not report or demonstrated important consequences of this procedure on the newborn [107], and thus far, the cost–benefit ratio seems reasonable. However, abnormal spermatozoa prepared for ICSI showed improper methylation patterns at imprinting regions, leading to conceive embryos affected by genetic imprinting disorders. Not surprisingly, the alterations in the sperm might explain why ICSI is the fertilization procedure that induced the highest number of differentially expressed genes [108].

An increased reported incidence of autism spectrum disorders in babies born from ART might find some possible source in epigenetic alterations [82]. Numerous studies have reported an increased risk of congenital imprinting disorders in children conceived by ART (Figure 2) [109,110]. In 2019, a wide epidemiological study in Japan found 3.44-, 4.46-, and 8.91-fold increased frequencies of Prader–Willi syndrome (PWS), Beckwith–Wiedemann syndrome (BWS), and Silver–Russell syndrome (SRS) after ART, respectively [111]. In particular, BWS, which is a clinically and genetically heterogeneous overgrowth disease, is caused by epigenetic defects at the 11p15 chromosomal region and a four- to six-fold increased risk of its outcome in ART pregnancies has been reported [22].

### 3.3. Immune Dysregulation at the Maternal-Fetal Interface

Increased immune activity at the maternal–fetal interface and significant histological and immunohistochemical differences were observed in placentas from pregnancies obtained by IVF of heterologous oocytes, compared to those obtained using homologous oocytes [112], and may be the consequence of a host versus graft rejection-like condition. This aspect was further investigated using 3D ultrasound analysis. This technique demonstrated a more marked reduction of first-trimester placental volume in pregnancies obtained using donor oocytes [113]. A recent retrospective cohort study, including 1114 singleton pregnancies, of which 105 conceived with IVF, further supports the involvement of the immune system in pregnancy complications associated with ART [114]. A higher incidence of villitis was observed in placentas from pregnancies obtained following IVF (16.2% vs. 8.3%; *p* = 0.007).

### 3.4. Mechanical Stress on Embryo and Placental Development (the Case of PGT-A)

PGT-A is an ART technique that is increasingly performed, considering that it represents 40% of all IVF cycles in the USA [115]. Its aim is to discover if produced embryos are aneuploid and, in this case, they should not be transferred into the uterus, in order to prevent implantation failure and miscarriages. PGT-A preliminarily requires the in vitro development of embryos up to the blastocyst stage, in order to collect 5 to 10 trophoblast cells. Embryos are then frozen while waiting for the genetic analysis results. Embryos with no or few aneuploidies are thawed and transferred (FET) in the uterine cavity after endometrial hormonal preparation using estrogen and progesterone. In this condition, natural ovulation and formation of the corpus luteum are suppressed. Similar to PGT-A, preimplantation genetic test for monogenic diseases (PGT-M) allows to screen for embryos affected by monogenic diseases.

Some authors have expressed concerns on the possibility that trophectoderm biopsy might disturb embryo development, leading to potential adverse consequences on obstetric outcomes [65,116]. It has been hypothesized that removal of cells from the tissue that will become the placenta does not affect embryo implantation rate but may negatively influence later phases of placentation. Placenta-derived anomalies, such as placenta previa and accreta, HDP, and FGR might be consequences of this abnormal placentation [117,118,119,120].

In a comparative study investigating obstetric and neonatal outcome following PGT-A in blastocyst-stage biopsy with frozen embryo transfer and cleavage-stage biopsy with fresh embryo transfer, Jing et al. (2016) found that PGT-A-FET was associated with a higher incidence of gestational hypertension (9.0% vs. 2.3%; *p* = 0.017) [121]. These data might indicate a higher risk of placental injury as a consequence of PGT-A procedure on the trophectoderm. Similar indications came from the results of a study on 345 singleton and 76 twin deliveries after PGT-M. PGT-M showed an increased risk of obstetric complications, when compared with pregnancies conceived spontaneously or by IVF without PGT [122]. In particular, these data showed a higher rate of HDP (6.9%), compared with spontaneous conception patients (2.3%) and the IVF group (4.7%). It was reported, as well, a higher rate of SGA neonates (12.4%), in comparison with those from spontaneous conception group (3.9%) and the IVF patients (4.5%). Moreover, Makhijani et al. (2021) observed a significantly higher probability of developing HDP in patients who underwent PGT, compared with those who did not (adjusted odds ratio (aOR) 1.943, 95% CI 1.072–3.521; *p* = 0.029) [123]. In this study, the potential influence of other factors causing HDP, such as the hormonal endometrial preparation was considered. In their binary logistic regression model, they found that, with the adjusted odds of HDP, the risk associated with trophectoderm biopsy remained significantly higher. A recent large single-center study found a two-fold higher risk of pregnancy-related hypertension following 241 blastocyst biopsy and frozen transfer than in 515 non-biopsied controls [123].

Conversely, is it worthwhile to look for embryonic aneuploidies. It was shown that, among embryos with aneuploid cells, 31% were of meiotic origin and 74% related to mitotic aneuploidies, with maximum aneuploidy originating by days 4–5 after fertilization, falling to 5–6% by day 7 [124]. Consequently, we should consider the environment influence on the mitotic origin of aneuploidies in developing embryos. From a strict clinical point of view, PGT-A does not improve IVF outcomes nor does it reduce miscarriage rates [125]. Moreover, healthy babies have been born after the transfer of aneuploid mosaic embryos [126,127,128]. This is not surprising since it is well known the self-correction ability of aneuploid embryos. It has been demonstrated that, in mice, a *p*-53-dependent autophagy-mediated apoptotic process is able to eliminate aneuploid cells [129]. In humans, this process is promoted by the bone morphogenetic protein-4 (BMP4) [130]. These mechanisms, together with the fact that the results obtained from a trophectoderm biopsy cannot be representative of the whole embryo [131], contradict the assumption that all aneuploid embryos should be eliminated and constitutes a strong criticism for the PGT-A clinical utility [125]. The Scientific and Clinical Advances Advisory Committee of HFEA recently changed the rating of PGT-A to “red”, meaning that there is no evidence to show that the treatment is effective and safe [125].

## 4. Metabolic and Cardiovascular Consequences of Placental Dysregulation in Mothers Following ART

Studies demonstrated that vascular dysfunctions in IVF pregnancies were similar to those identified when babies were conceived naturally from mothers affected by PE [132,133]. Sundheimer and Pisarska explain that the size of the placental bed and successful trophoblast invasion and spiral artery remodeling determine maternal blood flow. With these premises, abnormal placentation associated with infertility can represent a consequent marker of overall health for both the mother and her offspring [134]. It has been well known for a long time that inadequate trophoblast invasion and poor spiral artery remodeling may cause reduced vascular perfusion in pregnancy [135].

Women of advanced maternal age are generally at increased risk of GDM, HDP, operative and cesarean deliveries [136,137]; however, those who conceive with the use of IVF are at increased risk of retained placenta, suggesting that placentation abnormalities may contribute to maternal morbidity, and this may be more pronounced in women with infertility [138].

Fertility treatments (IVF and NON-IVF combined) have been associated with an increased risk of severe maternal morbidity (SMM), defined as unexpected outcomes of labor and delivery that result in significant short- or long-term consequences to a woman’s health after [139]. In a population-based cohort study of 114,409 singleton pregnancies with conceptions dating from 11 January 2013 to 10 January 2014 in Ontario, IVF was associated with an increased risk of SMM (rate 11.3/1000; aRR 1.89, 95% CI: 1.06–3.39) [140]. In this study, the authors also noted supra-additive effects of high body mass index (BMI) and IVF on the risk of PE and GDM. The link between fertility treatment and SMM was also confirmed in another cohort study conducted from Ontario between 2006 and 2012, demonstrating that women undergoing fertility treatment are at higher risk of SMM with a relative risk of 1.39 (95% confidence interval (CI) 1.23–1.56). Indicators of SMM were considered severe postpartum hemorrhage, maternal admission to an intensive care unit, puerperal sepsis, hysterectomy and cardiac conditions [141]. The American Heart Association considers a history of PE or HDP as a major risk factor in these women for the development of cardiovascular diseases (CVD) [142].

In summary, cardiovascular and metabolic diseases derived from impaired placentation following infertility treatments may have long-term consequences on both the mother and the newborn.

## 5. Health Risk in Infancy as a Consequence of Placental Dysfunction Following ART

According to the “developmental origins of health and disease theory” [143] and the Barker hypothesis of the developmental origins of chronic adult disease [144], placental dysfunction and abnormal fetal development may have long-term consequences on the neonate and his development, from childhood to adult life. A higher risk of diabetes mellitus and CVD were found in children whose mothers had PE and HDP, by long-term follow up observations [145,146]. A track of this condition is represented by the finding of reduced endothelial function in mothers and children after PE [147].

Evidence suggests, at least in animal studies, that IVF can promote endothelial dysfunction with increased vessel stiffness in offspring. This can induce arterial hypertension in vivo and an epigenetic origin of the condition has been suggested [132]. IVF causality is also suggested by the significantly elevated activity of enzymatic regulators in the cardiovascular system of IVF offspring [148]. Additionally, there is increasing evidence that offspring conceived by IVF displays a level of vascular dysfunction similar to that seen in children spontaneously conceived by mothers with PE [132,133]. Of note, in 2015 in a randomized case-control trial, antioxidant administration to IVF children was able to improve NO bioavailability and responsiveness of both systemic and pulmonary circulation [149], thus indicating that vascular dysfunctions induced by ART are reversible in young people.

Regarding the increased metabolic risk later in adult life, IVF-conceived mice showed decreased glucose tolerance, increased fasting glucose levels, and reduced insulin-stimulated protein kinase B (Akt) phosphorylation in the liver later in their adult life [150]. These findings are similar to those seen in humans. Some individuals conceived by ART have shown LBW, higher weight, height, and BMI in late infancy [151] and fasting insulin levels [152]. Adolescents conceived by ART showed significant differences in growth kinetics, glucose levels, and blood pressure in comparison with those conceived naturally by sub-fertile parents [153,154], suggesting that these alterations may be caused by ART procedures. Development delay at birth (probably caused by epigenetic alterations) and accelerated growth in late infancy, with metabolic alterations in adolescence, may predispose these individuals to chronic diseases such as obesity and type 2 diabetes [155,156]. An epigenetic programming of metabolism during prenatal and postnatal periods as a response to imprinting alterations that occurred during early embryonic development has been hypothesized [156,157].

## 6. Simultaneous Action of Factors Dysregulating Normal Placentation

Infertility and its treatments, including ART and its various procedures (ICSI, blastocyst culture + FET + PGT-A with ovarian stimulation or hormonal preparation), may individually or synergistically participate in the dysregulation of embryo and placenta development. Consequently, it is probably impossible to separate the relative contribution of the many factors influencing embryo, placental, and newborn health when an infertile woman is treated with the many types of ART techniques. These techniques may represent confounding factors for a full understanding of previous studies and future ones to be designed.

Each technique may lead to important epigenetic changes and differential gene expression in the placenta [82,83] or damage the developing embryos with thermal, oxidative and mechanical stressing actions [82,98,158].

Pivotal reproductive hormones, such as human chorionic gonadotropin, progesterone and estradiol, are found at high concentrations at the maternal–fetal interface during vessel remodeling. This observation allows to hypothesize that such relevant vascular transformation may be under the influence of these hormones, which may control trophoblast migration [90,159]. A potential role in placental function dysregulation has been proposed for estrogens. In this respect, elevated estrogen exposure (as in controlled ovarian hyperstimulation) has been associated with higher rates of LBW and FGR [160].

Are perinatal adverse outcomes in infertile patients the consequence of ART or are they the consequence of the underlying infertility? A possible answer to this basic question may come from infertile couples conceiving spontaneously with no ART treatments; in these couples, a higher risk of PTB and LBW has been reported [1]. However, an increasing number of observations show vascular dysfunctions similar to those observed in PE in IVF offspring [132,133], and a common mechanism of action could be postulated.

The induction of defective methylation and consequent alteration of gene expression, which may impair placentation [85], can be caused by oxidative stress [161,162], as in endometriosis and by the altered hormonal milieu, i.e., supraphysiologic estradiol levels due to ovarian hyperstimulation [97]. Particular negative effects on embryo development may occur in PGT-A procedures, in which the epigenetic risk for long in vitro culture may be added to the trophectoderm mechanical stress. If the patient is also affected by PCOS, this adverse metabolic condition for the placenta would considerably increase the overall obstetric, perinatal and postnatal final risk [76,77]. All these factors could exert additive effects, leading to the pathologic condition.

## 7. Final Remarks

The huge and increasing number of ART cycles in the world, together with the discovery of epigenetic, obstetric, maternal and newborn associated risk, should raise concerns in the medical and scientific communities. Observing the three main world ART registers (United States of America, Europe, Australia and New Zealand) during the period 1997–2016, we acknowledge that the numbers of recorded ART treatments increased considerably (5.3-fold in Europe, 4.6-fold in the USA, 3.0-fold in Australia and New Zealand [163]. In 2016, the ICSI technique overcome IVF and accounted for 50% to 60% of all cycles of in vitro insemination. A sharp rise in the number of freeze-all cycles has been observed both in Australia and New Zealand (reaching 26.5% of all oocyte retrieval cycles for IVF and ICSI in 2016) and in the USA (19.2% in 2016). It is relevant to highlight that the majority of the freeze-all cycles involve blastocyst stage embryos. Practically, FET has overcome fresh embryo transfer in the USA and Australia. PGT-A in the USA is performed in 40% of IVF cycles with an increasing trend [115] and, in Australia, represents the fourth most used type of ART technique [163]. This context should be considered when trying to investigate related and prospective ART risks in a growing infertile population. From a clinical point of view and according to the presented data, we can imagine an increasing risk with this pattern: ovarian stimulation < IVF < ICSI < Blastocyst culture < PGT-A. On this basis, we could speculate the higher risk in a patient with PCOS or endometriosis that performs PGT-A.

Considering the many factors and metabolic pathways that we are continuously discovering to be involved in reproductive processes, from fertilization to embryo implantation and feto-placental development, we are far from completing the entire and extremely complex picture. Our experimental findings are able to shed light on small and specific tiles of the complex mosaic regarding many reproductive patterns, often not well known or even completely obscure to our understanding and knowledge.

These observations open the question that regard our medical action, that is, the prevention of consequences linked to artificial procedure that move too far away from physiologic patterns. Not completely knowing the interrelated quite complex molecular pathways skipped or artificially modified, we cannot pharmacologically correct them in a more rational and appropriate way.

With these premises, the prevention of impaired placentation might be considered before starting an ART procedure with a metabolic improvement to women (e.g., dietary and pharmacologic tools in case of PCOS and insulin resistance); additionally, a secondary prevention might be represented by the right choice of therapeutic techniques and protocols (e.g., less invasive embryo culture/procedures and mild stimulation) and with a more personalized obstetric control before pathologic conditions of the placenta become clinically evident as hypertensive or metabolic disorders. If pathologic placentation produces detectable effect starting on the fifth or sixth gestational week, we should not wait until the second or third trimester to observe its clinical consequences, and first trimester miscarriages might not be of genetic origin. A higher prevalence of spontaneous abortion with ART should prompt us to investigate both embryo aneuploidies and placental pathology [164].

In conclusion, it is reasonable to continue research on all key points of the reproductive process, and it is logical to try to correct or prevent (using medical procedures) what is becoming pathologic on the basis of our increasing knowledge. However, maybe it is wiser not to stray too far from the physiology of reproduction. Those who are dealing with ART are requested to study with more interest the physiology and the pathology of the placenta, not only from the usual obstetric point of view. Balancing the pro and cons of our reproductive interventions and observing long-term health of the mother and offspring, remain important topics, although much has to be investigated on biological and clinical levels. With this aim, collaborative studies between clinicians and biologists should be strongly encouraged.

## Data Availability

Not applicable.

## Abbreviations

PTB	preterm birth
LBW	low birth weight
PE	preeclampsia
FGR	fetal growth restriction
PCOS	polycystic ovary syndrome
GDM	gestational diabetes mellitus
ART	assisted reproductive technology
IVF	in vitro fertilization
ICSI	intracytoplasmic sperm injection
FET	frozen embryo transfer
OD	oocyte donation
PGT-A	preimplantation genetic testing for aneuploidy
HDP	hypertensive disorders of pregnancy
VLBW	very low birth weight
PlGF	placental growth factor
sFLT-1	soluble fms-like tyrosine kinase 1
EGFR	epidermal growth factor receptor
sENDOGLIN	soluble endoglin
EGFL7	epidermal growth factor-like domain 7
TGF-β	transforming growth factor-beta
NO	nitric oxide
TNF-α	tumor necrosis factor-alpha
IL	interleukin
SGA	small for gestational age
LGA	large for gestational age
TH	thyroid hormone
LT4	levothyroxine
PIH	pregnancy induced hypertension
PGT-M	preimplantation genetic test for monogenic diseases
BMP-4	bone morphogenetic protein-4
CVD	cardiovascular disease
PWS	Prader–Willi syndrome
AS	Angelman syndrome
BWS	Beckwith–Wiedemann syndrome
SRS	Silver–Russell syndrome
SMM	severe maternal morbidity
